# Mirror Box Training in Hemiplegic Stroke Patients Affects Body Representation

**DOI:** 10.3389/fnhum.2017.00617

**Published:** 2018-01-04

**Authors:** Giorgia Tosi, Daniele Romano, Angelo Maravita

**Affiliations:** ^1^Department of Psychology, Università degli Studi di Milano-Bicocca, Milan, Italy; ^2^NeuroMi—Milan Center for Neuroscience, Università degli Studi di Milano-Bicocca, Milan, Italy

**Keywords:** body schema, mirror box, body representation, hemiplegia, stroke

## Abstract

The brain integrates multisensory inputs coming from the body (i.e., proprioception, tactile sensations) and the world that surrounds it (e.g., visual information). In this way, it is possible to build supra-modal and coherent mental representations of our own body, in order to process sensory events and to plan movements and actions in space. Post-stroke acquired motor deficits affect the ability to move body parts and to interact with objects. This may, in turn, impair the brain representation of the affected body part, resulting in a further increase of disability and motor impairment. To the aim of improving any putative derangements of body representation induced by the motor deficit, here we used the Mirror Box (MB). MB is a rehabilitative tool aimed at restoring several pathological conditions where body representation is affected, including post-stroke motor impairments. In this setting, observing the reflection of the intact limb in the mirror, while the affected one is hidden behind the mirror, can exert a positive influence upon different clinical conditions from chronic pain to motor deficits. Such results are thought to be mediated by a process of embodiment of the mirror reflection, which would be integrated into the representation of the affected limb. A group of 45 post-stroke patients was tested before and after performing a MB motor training in two conditions, one with the mirror between the hands and one without it, so that patients could see their impaired limb directly. A forearm bisection task, specifically designed to measure the metric representation of the body (i.e., size), was used as dependent variable. Results showed that, at baseline, the forearm bisection is shifted proximally, compatibly with a shrink of the metric representation of the affected arm towards the shoulder. However, following the MB session bisection scores shifted distally, compatibly with a partial correction of the metric representation of that arm. The effects showed some variability with the laterality of the lesion and the duration of the illness. The present results call for a possible role of the MB as a tool for improving altered body representation following post-stroke motor impairments.

## Introduction

The body holds an accurate brain representation of its anatomical components, which includes the actual position and reciprocal spatial relations of the limbs and their segments (Maravita et al., 2003; Vallar and Maravita, 2009; Berlucchi and Aglioti, 2010; Sposito et al., 2012).

We constantly receive many different inputs from either the world outside the body or the body itself, that the brain integrates in order to create a supra-modal and coherent mental representation of our own body (Berti, 2013). In the attempt to theorize the nature of body representation, alternative cognitive models have split this concept in different components trying to outline their functions (Schwoebel and Coslett, 2005; Carruthers, 2008; de Vignemont, 2010; Longo et al., 2010). The body schema is the component on which most authors agree and is defined as a dynamic sensorimotor representation that guides perception and action. Body schema is supposed to be mostly out of awareness contributing to body-space interactions in an automatic fashion (Dohle et al., 2004; Haggard and Wolpert, 2005; de Vignemont, 2010; Longo et al., 2010). Other levels of body representation are not used primarily for action and are believed to act at a more conscious level, but their taxonomy is still debated (de Vignemont, 2010; Longo et al., 2010). According to Longo et al. (2010), three levels would be in play. The first, named somatoperception, would allow perceiving the body in a first-person perspective and would gather information from a postural schema, i.e., a dynamic representation of the position of the body in space. A second level, named superficial schema, would provide the localization of somatic sensations on body surface. The third level, named body metric, would be a representation of body parts configuration.

One important thing that seems to be shared by different aspects of body representation is their plastic nature. Cortical plasticity is a fundamental feature of the nervous system, ensuring constant adaptive changes for behavior (Kolb et al., 2003). Body representation is also constantly updated due to both long-term processes, such as development and skill learning, and short-term events, such as single movements. Remarkably, not only positive, but also negative plastic changes can occur in the brain following disease and, crucially for the purpose of the present article, following body part disuse and immobilization. This is relevant given that motor impairments, including hemiparesis and hemiplegia, are the most common deficits after stroke (Schaechter, 2004). Although arm training positively affects brain plasticity and may have positive effects on recovery (Nelles et al., 2001; Dohle et al., 2009), rehabilitation of motor skills is often incomplete. Furthermore, progressive brain plasticity can also occur, leading to a reduction of cortical representations of motor areas (Flor et al., 2006; Bassolino et al., 2015). Critically, such changes go beyond the area directly affected by the stroke (Hallett, 2001).

Indeed, motor impairments, preventing the regular and active use of the affected limbs, lead to the decrease of cortical representations of sensorimotor areas of those body parts (Dohle et al., 2009), by further affecting limb functionality. This corresponds to the idea of “learned paralysis”, first introduced by Taub et al. (1998) who studied the motor behavior of surgically deafferented monkeys. The concept of learned paralysis was extended to humans by Ramachandran (1993). He suggested that the visual feedback of immobility of the affected limb following motor output, would progressively reinforce, in the patient, the acquired knowledge that the limb cannot move. As a consequence, the representation of that limb in the sensory cortex, lacking sensory input, would progressively shrink. Concurrently spared neurons would be functionally reorganized, increasing the representations of the adjacent body parts (Hallett, 2001). These rearrangements result in increased disability and enhance the clinical effects of motor impairment.

In the present article, we sought for behavioral evidence of such a deranged brain representation of the affected limb in stroke patients with motor impairment. Moreover, we tested whether a training with the Mirror Box (MB) could positively affect this condition. The MB has been successfully used to improve the performance of patients affected by motor impairments after stroke (Altschuler et al., 1999; Yavuzer et al., 2008; Michielsen et al., 2010). First described in the treatment of phantom limb pain (Ramachandran et al., 1995), MB typically consists of a vertical mirror positioned along the midsagittal plane of the patient’s body, in the center of a box. The patient places his affected limb/stump on one side of the box, behind the mirror, and the healthy one in the other side of the box, facing the mirror. With this arrangement, the reflection of the healthy arm visually mimics the shape and location of the hidden, affected one. Then the patient is asked to try and perform bilateral movements with the affected and the non-affected limbs at the same time. This procedure typically generates a subjective sensation of both limbs moving, even if the affected one cannot move. If the procedure is repeated for a number of sessions it may lead to some degree of improvement of motor performance (see Ramachandran and Altschuler, 2009 for a review).

The working hypothesis of the present article is that the embodiment of the mirror-reflected hand would enhance the reorganization of body representation in hemiplegic patients (Medina et al., 2015). Embodiment corresponds to a specific type of information processing. de Vignemont (2011) proposed the following definition: “(an object) E is embodied if and only if some properties of E are processed in the same way as the properties of one’s own body”. Several authors (Romano et al., 2013; Medina et al., 2015) reported this process as the underlying mechanism of the MB, which operates by updating the body representation through the embodiment of the mirror-reflected body part. In particular, we searched for an effect of the MB over the metric representation of the affected arm (Longo et al., 2010) which may be impaired by the lesion, and contribute to the motor deficit. To test this idea, we used the forearm bisection task, a paradigm proved to be effective in measuring the metric representation of the body (Sposito et al., 2010). In this task participants are required to indicate the midpoint of one of their forearms through a ballistic movement performed with the opposite hand. Quick and uncorrected movements are requested in order to maximize the effect of the current, inner representation of the body. Ballistic movements allow to minimize the impact of any tactile, visual or proprioceptive references coming from the environment or the arms. This task has attested the superiority of body metric representations as compared to extrapersonal objects. Sposito et al. (2010) found smaller errors when participants bisected their own forearm as compared to a cylinder occupying the same space sector. This difference was even more pronounced for neglect patients in whom the rightward bisection bias, which is a marker of a neglect condition, was reduced in the forearm as compared to solid object bisection. In another study, forearm bisection proved to be sensitive to body representation changes following tool use. In healthy participants, reaching for objects in far space through a rake induced a distal shift in forearm bisection (Sposito et al., 2012) compatibly with the putative extension of body representation towards the tip of the tool, following its skilled use (Maravita and Iriki, 2004). Additionally, in a group of patients showing the pathological behavior of feeling a sense of ownership towards alien limbs, it was shown a distal bias of forearm bisection following the mere observation of tool-reaching, when performed by the self-misattributed alien limb (Garbarini et al., 2015).

Here, first we assessed whether hemiparetic post-stroke patients show any proximal bias the forearm bisection task of the affected limb in a group of, likely due to non-use. Critically, we investigated if this bias may change after a MB training session, compared to a control training without the mirror. In addition, we aimed at qualifying any effects of the MB relatively to individual clinical features, such as lesion side and the duration of the disorder. We chose to evaluate metric representation of the distal portion of the paretic arm (i.e., from the elbow to the tip of the middle finger), which is typically the most affected part as compared to more proximal segment (Hallett, 2001).

## Materials and Methods

### Participants

A continuous series of 53 participants was selected to take part in the study (15 female, 38 male). Patients with acquired motor impairment after brain injury were recruited at the Rehabilitation Unit of Bassini Hospital, Cinisello Balsamo (Mi), and Sant’Antonio Abate Hospital, Somma Lombardo (Va). Inclusion criteria were: (i) preserved cognitive functions for understanding the assessment and rehabilitative tasks; (ii) single, unilateral brain lesion affecting strength of the upper limb. Exclusion criteria included: (i) worsening of health condition between the two experimental sessions; (ii) cerebellar lesions; (iii) multiple brain accidents; and (iv) orthopedic or musculoskeletal disorders affecting upper limbs. Due to exclusion criteria, the final sample of the study was composed by 45 patients (15 female; mean age = 66.36 ± 11.81 [range 38–87]; mean education = 9.42 ± 4.08 [range 3–18]). 25 patients were in the subacute phase, i.e., within 3 months from the stroke event (range 1–3 months) and 20 held chronic impairment (range 4 months–10 years; Yavuzer et al., 2008). Concerning the lesion side, there were 22 left brain damage (LBD) and 23 right brain damage (RBD) patients.

Essential biographic and clinical characteristics of participants are presented in Table 1.

**Table 1:** Demographic characteristics of the sample.

Patient	School age	Lesion side	Chronicity
P1	8	L	no
P2	13	L	no
P3	8	R	yes
P4	13	R	no
P5	8	L	no
P6	5	L	yes
P7	5	R	no
P8	11	R	no
P9	12	R	no
P10	5	L	yes
P11	13	L	yes
P12	13	R	yes
P13	5	R	yes
P14	5	R	no
P15	8	R	no
P16	7	R	yes
P17	5	R	no
P18	11	S	no
P19	8	L	no
P20	10	L	yes
P21	5	R	no
P22	3	L	yes
P23	5	L	no
P24	5	L	yes
P25	13	R	yes
P26	6	L	yes
P27	5	R	no
P28	13	L	yes
P29	9	L	yes
P30	16	R	no
P31	13	R	no
P32	5	L	no
P33	10	R	no
P34	8	R	no
P35	5	L	no
P36	8	R	yes
P37	17	R	yes
P38	13	L	no
P39	8	R	yes
P40	13	R	no
P41	11	R	no
P42	18	L	yes
P43	8	L	yes
P44	16	L	yes
P45	18	L	yes

***Lesion side**: L = left hemisphere, R = right hemisphere; **Chronicity**: yes = stroke event occurred more than 3 months before (range 4 months–10 years), no = stroke event occurred within 3 months from the stroke event (range 1–3 months)*.

All subjects gave their informed oral consent previous to testing, in accordance with the Declaration of Helsinki (World Medical Organization, 1996). Motor trainings were administered as a part of the rehabilitation program, to which patients have given their written consent to the hospital. The protocol was approved by the Ethic Committee “Commissione per la Valutazione della Ricerca, Dipartimento di Psicologia” of the University of Milano-Bicocca.

### Neuropsychological Screening

All patients underwent a modified version of the standard neurological examination by Bisiach et al. (1983, 1986), featuring the testing of: strength, visual field, proprioception, tactile perception and personal neglect. The custom procedure and scoring for each specific test is detailed below.

#### Strength

Participants had to extend both their arms in front of them, keeping their palms up, with closed eyes. The scores were: 0 = patient keeps the position at least for 30 s; 1 = patient shows finger abduction, main creuse (i.e., thumb abduction), Gierlich (i.e., hand pronation), Barrè (i.e., downward drift of the limb) without touching the table within 15 s; 2 = Barrè sign, touching the table within 15 s; 3 = Barrè sign, touching the table within 5 s.

#### Visual Field Examination

A baseline set of 20 randomized visual stimuli was delivered to the patient’s upper visual field, comprising: four ipsilesional stimuli, i.e., delivered to the same side as the cerebral lesion, six contralesional stimuli, i.e., delivered to the side opposite to the lesion, and 10 bilateral stimuli. If patients got seven or less contralesional stimuli overall (i.e., among contralesional and bilateral trials), then 12 further ipsilesional and 10 contralesional randomized stimuli were presented. The scores were: 0 = patient gets at least 8/10 bilateral stimuli and 10/14 ipsilesional; 1 = patient gets ≤7 bilateral stimuli with 10/14 ipsilesional; 2 = patient gets 4/7 unilateral contralesional stimuli with 10/14 ipsilesional; 3 = patient gets 0/3 unilateral contralesional stimuli with 10/14 ipsilesional.

#### Tactile Perception

Twenty randomized tactile stimuli (4 ipsilesional, 6 contralesional, 10 bilateral) were applied on the dorsal surface of the patient’s hands. If the patient, blindfolded, got seven or less contralateral stimuli, then 12 further ipsilesional and 10 contralesional randomized stimuli were presented. The scores were: 0 = patient gets 8/10 bilateral stimuli with 10/14 ipsilesional ones; 1 = patient gets ≤7 bilateral stimuli with 10/14 ipsilesional ones; 2 = patient gets 4/7 unilateral contralesional stimuli with 10/14 ipsilesional ones; 3 = patient gets 0/3 unilateral contralesional stimuli with 10/14 ipsilesional.

#### Proprioception

The examiner moved the patient’s impaired hand and the subject had to move the other arm in the same way, with closed eyes. The scores were: 0 = correctly done; 1 = the patient perceived the movement, but could not detect the hand’s final position; 2 = the patient did not perceive the movement.

#### Personal Neglect

Patient sat on a chair, with his/her forearms radially extended on a table and closed eyes. The subject was asked to reach and touch his/her plegic hand with the healthy one. The same task was repeated with the experimenter’s hand between the patient’s ones, in order to test for any mistakes in reaching behavior (misattribution). The scores were: 0 = (absent) patient correctly reaches his/her impaired hand in both conditions; 1 = (present), patient cannot reach his/her impaired hand in at least one condition.

The procedure followed the standard method described by Bisiach et al. (1983, 1986).

### Experimental Procedures

Patients underwent two experimental sessions, during which two different motor training conditions were tested, either using the MB or not. Training weeks were intermingled by one wash-out week and the order of trainings (MB/no-MB) was counterbalanced across participants. The overall design resulted in a 2 × 2 crossover design (AB/BA): 23 patients used the MB during the first session, while the other 22 started with the training without the MB (see below).

In each session patients performed two forearm bisection tests, one before and one after the motor training.

#### Forearm Bisection

Patients sat on chair, with their forearms placed radially on a table. They were asked to indicate with their index finger the midpoint of their impaired arm, considering the tip of the middle finger and the elbow (olecranon) as the distal and proximal extremes. Patients were requested to indicate the midpoint with a ballistic movement, with closed eyes. On each trial, a flexible ruler was used to measure the patient’s performance, setting the 0-cm point in correspondence of the tip of the middle finger. 15 trials of bisection pointing were recorded. Before starting the task, the arm length was measured.

There was no time constraint for the bisection task, but corrections were not allowed. Patients performed a total of 30 trials (15 before and 15 after each training) in every session, for a total of 60 trials per participant.

A percentage score was calculated for each forearm bisection trial using the following formula: [(p/arm length)*100], where p indicates the subjective midpoint, measured with a flexible ruler on each trial. In this formula, a value of 0% corresponds to the tip of the middle finger, 100% corresponds to the elbow. A value higher than 50% indicates a deviation of the subjective midpoint towards the elbow, i.e., proximal deviation, while a value lower than 50% indicates a deviation towards the hand, i.e., distal deviation (Sposito et al., 2012; Garbarini et al., 2015). For the analysis, we considered the difference between pre- and post-training bisections (pre-training minus post-training). The shift obtained with this formula, was positive in case of distal deviation and negative for proximal deviation.

#### Motor Training

The training, lasting 10 min, consisted of simple hands movements (e.g., opening/closing the hands, whole hand tapping, single finger tapping, etc., see Table 2) that patients were invited to try and perform with both hands simultaneously, no matter the motor performance of the affected hand (Altschuler et al., 1999; Ramachandran and Altschuler, 2009).

**Table 2:** Hand movements requested during 10 min of motor training, with or without the Mirror Box.

Hand movements	Duration
Opening/closing the hands	2 min
Whole hand tapping	2 min
Single finger tapping	2 min
Whole hand lateral rotation	2 min
Tapping of palm and back of the hand alternatively	2 min

We chose to train only the hand, and not the whole arm, because motor impairments typically affect more the distal than the proximal limb region, thus leading to a reduction in motor abilities and cortical representation of the former, with relatively retained strength of the latter (Dohle et al., 2009). Since there is a “functional competition” between body parts for cortical representation in the motor region, it is possible that simultaneous use of both proximal and distal segments of the limb may reduce the chance for the more affected hand muscles to improve their representations (Hallett, 2001).

**Figure 1:**
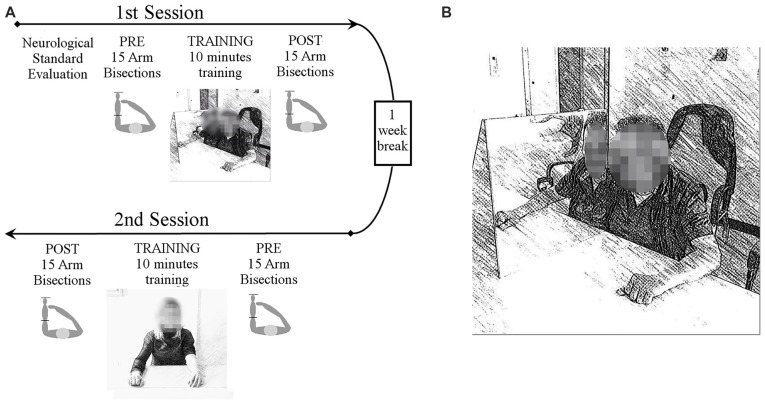
Experimental procedure **(A)**. All patients underwent a standard neurological examination, including strength, visual field, proprioception, tactile perception and personal neglect. Then patients underwent two experimental sessions, separated by 1 week, in which two different motor trainings were tested, either using the mirror box (MB) or not. In each session participants performed two forearm bisection tests, one before and one after each motor training. The training lasted 10 min and it consisted of simple hands movements that patients were invited to try and perform with both hands simultaneously, with or without the MB. Training sessions order was counterbalanced across participants. Experimental set-up of the MB training **(B)**. The MB was placed with the reflective surface parallel to the participant’s midsagittal plane. The impaired limb was placed inside the MB and was hidden from view, while the contralateral limb was placed in front of the mirror in such a way that its reflection exactly matched the felt position of the hand inside the MB.

Each patient performed the motor training with and without the MB in two different sessions (see Figure 1A for a summary of the experimental procedure). The MB consisted of a triangular-shaped structure placed in front of the patient. The box had the basis and one face made of plywood (50 × 50 cm and 50 × 75 cm), while the third face was a mirrored surface (50 × 50 cm).

In the MB condition the experimental device was placed with the reflective surface alongside the participant’s midsagittal plane. The impaired limb was inside the MB and hidden from view, while the contralateral limb was located in front of the mirror. Hands were placed at the same distance from both side of the mirror, in the same position. With this arrangement, participants could see the hand outside the box through its mirror reflection, that exactly matched the felt position of the hand inside the box (Figure 1B). Attention toward the mirror reflection was constantly encouraged by the experimenter. In the control condition patients were asked to perform the same actions without the MB, so to leave both hands visible.

### Analysis

We used a Linear Mixed Model (LMM) approach as implemented in SPSS 22.0 (IBM, Chicago, IL, USA) that is a more powerful and flexible method as compared to traditional ANOVA. We thus run LMM, three-way ANOVA with the bisection shift (i.e., the difference between pre- and post-training bisection percentage scores) as dependent variable. Fixed effect factors were condition (MB/no-MB), as within-subject factor, and lesion side (right/left) and chronicity (subacute/chronic) as between-subject factors. Patients were considered as a random effect variable, in order to properly use all observations for effect estimation. We considered 95% Confidence Interval (CI), in order to explore interactions reliably, without running additional *post hoc* tests (Cumming, 2014).

## Results

The main factor condition was significant (*F*_(1,1304.6)_ = 28.385; *p* < 0.001), showing that MB training positively affects bisection task. We found a distal shift in the MB condition (CI: 0.7; 3.4), while a proximal deviation resulted following the no-MB condition (CI: −1.5; 1.1).

Moreover we found a significant interaction between condition and lesion side (*F*_(1,1304.6)_ = 4.910; *p* < 0.05). Right brain damaged patients showed a distal shift both in the MB (CI: −0.4; 3.4) and in the no-MB (CI: −1.7; 2.0) condition, even if the deviation is greater in the first condition. On the contrary for left brain damaged patients we found a distal shift only with the mirror (CI: 0.7; 4.4), while the bisection point shifts toward the shoulder in the no-MB condition (CI: −2.5; 1.2).

Crucially we found three-way significant interaction involving all the fixed effects, i.e., condition, lesion side and chronicity (*F*_(1,1304.6)_ = 11.128; *p* = 0.001). In the MB condition a larger distal shift was observed in chronic left brain damaged patients (CI: 1.3; 6.0) than right brain damaged patients (CI: −2.0; 4.0). In subacute patients, the average distal shift is comparable following left (CI: −1.3; 4.4) and right (CI: −0.2; 4.2) brain damage.

**Figure 2:**
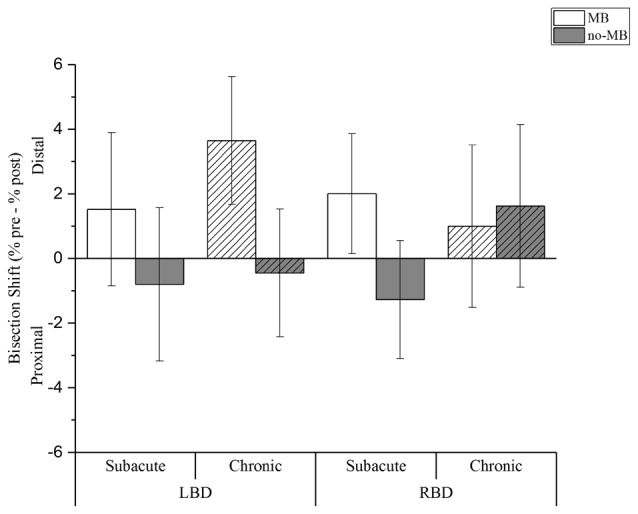
Experimental results. Columns indicate the shift (%) of the perceived forearm midpoint calculated as pre-training performance minus post-training performance. Positive values indicate a shift of perceived midpoint towards the hand (i.e., distal deviation), negative values indicate a shift of perceived midpoint towards the elbow (i.e., proximal deviation). Light columns show the results for the MB training condition, dark columns for the no-MB training condition. Full color columns show results for subacute groups, diagonal lines pattern columns represent the chronic groups. Thin bars indicate Confidence Intervals (CIs) set at 95% level.

Conversely, we found a proximal shift in the no-MB condition in most of the patients (subacute left brain damaged (CI: −3.6; 2.0); chronic left brain damaged (CI: −2.8; 1.9); subacute right brain damaged (CI: −3.5; 0.9)), except for chronic right brain damaged that showed a deviation towards the hand (CI: −1.4; 4.6) in this condition too (see Table 3 and Figure 2).

**Table 3:** Mean percentage score at the bisection task pre-training and post-training in all patients’ groups.

		MB		no-MB	
		Pre- training	Post-training	Pre- training	Post-training
**LBD**	Subacute	63.4 ± 3.1	61.8 ± 3.1	63.9 ± 3.1	64.7 ± 3.1
	Chronic	69.2 ± 2.6	65.5 ± 2.6	67.4 ± 2.6	67.8 ± 2.6
**RBD**	Subacute	70.2 ± 2.4	68.7 ± 2.4	67.1 ± 2.4	68.1 ± 2.4
	Chronic	66.4 ± 3.3	65.4 ± 3.3	66.8 ± 3.3	65.1 ± 3.3

The score was calculated as follows: [(p/arm length)*100], where p is the indicated midpoint. 0% = tip of the middle finger, 100% = elbow (Olecranon). A score > 50% indicates a deviation towards the elbow (proximal shift); a value < 50% indicates a deviation towards the hand (distal shift).

No further significant effects emerged (all other *p*-values > 0.210).

## Discussion

Body representation, a supra-modal and coherent mental schema of our own body (Berti, 2013), is largely influenced by sensory function, vision, actions and movements (Maravita et al., 2003; Carruthers, 2008; Cardinali et al., 2009; Longo et al., 2010; Sposito et al., 2010). In particular, motor and biomechanical constraints critically segment our body into prototypical functional units (de Vignemont et al., 2007). These components, through their ability to move and to perceive a movement, can affect the global body representation. When a brain injury damages central structures, devoted to planning and controlling movements, motor functions are significantly affected. Moreover, the non-use of the affected limb further impinges on the motor deficit by adding what has been called “learned paralysis” (Taub et al., 1998). This additional deficit has been proposed to be grounded in the progressive reduction of the cortical representation of that underused body part, representing a form of maladaptive plasticity (Dohle et al., 2009). The facilitation of motor function induced by the MB may potentially help to improve the cortical representation of the affected body part, thus contrasting the effects of negative plasticity. This mechanism has been already proposed to be effective in the treatment of phantom limb pain with the MB, as originally described by Ramachandran and Rogers-Ramachandran (1996). While MB was already proven to be effective in rehabilitating motor functions (Altschuler et al., 1999; Yavuzer et al., 2008; Ramachandran and Altschuler, 2009), our experiment shows for the first time that MB could also induce changes in body representation among patients with post-stroke motor deficit.

Although the MB is a promising rehabilitative tool, its underlying mechanisms in improving hemiparesis still need to be clarified. Recent experiments in neurologically unimpaired participants point to the importance of the embodiment of the reflected limb’s image in affecting sensory processing of the hidden arm (Longo et al., 2009; Romano et al., 2013). Embodiment can be defined as the attribution of some properties of an external object to one’s own body (de Vignemont, 2011). In this theoretical framework, MB would create a contrast between the perception of an impaired arm and a visual feedback compatible with a movement performed by that arm. In this conflicting situation, the visual capture from the reflected image would overwrite, to some extent, proprioceptive and somatosensory information. It would result into the embodiment of the mirror-reflected hand together with its properties of a functional limb (Romano et al., 2013). This illusory representation of an efficient limb, would then be used to plan movements (Garry et al., 2005; Holmes et al., 2006) and process sensory input like visual (Ritchie and Carlson, 2010), proprioceptive (Romano et al., 2013) and tactile (Bultitude et al., 2016) information. Neuroimaging results in stroke patients affected by motor impairments seem to corroborate this view by showing that a MB setting, analogous to the one used in the present work, activates regions of the precuneus and posterior cingulate cortex, which are critical for body and spatial representations (Michielsen et al., 2011). The occurrence of embodiment has been shown in different populations, such as healthy people (Romano et al., 2013) and patients with brain damage (Garbarini et al., 2015). Moreover, an interesting suggestion about the relationship between embodiment and cortical plasticity comes from the studies on spinal cord injury (Lenggenhager et al., 2012; Scandola et al., 2014). In particular, Lenggenhager et al. (2012) pointed out that plasticity-related cortical reorganization induced by spinal cord injury could impacts on the sense of body ownership and the effectiveness of bodily illusions. A similar cortical reorganization of the underused body part could be in play in our patients and was likely influenced by MB training (Medina et al., 2015).

The present work supports the idea that MB could exert a direct effect on subjective representation of the body. Particularly in order to measure the subjective representation of the impaired limb, we employed a forearm bisection task in which patients were asked to point at the perceived midpoint of their damaged limb. In order to do that, they implicitly needed to mentally represent the length and the position of the to-be-bisected body part (i.e., the impaired arm in our case). This paradigm has been used in investigating the metric representation of the body in healthy participants and brain-damaged patients (Sposito et al., 2010). Moreover, it has been proved to be sensitive to experience-induced plasticity, such as that induced by a short training with a tool (Sposito et al., 2012; Garbarini et al., 2015).

Our results showed that forearm bisection task is selectively modulated by the MB training and not after an unspecific motor training without the MB. These findings suggest that bilateral movements of the hands are not enough to induce a recalibration of body metric representation, ruling out a possible general effect of the intention-to-move.

It is worth noting that all patients showed a proximal deviation of the subjective midpoint at baseline of about 17%, suggesting that the arm was implicitly represented shorter than it actually was. This is in contrast with previous experiments using a similar forearm bisection task in neurologically unimpaired participants, where the baseline deviation was about 3% toward the hand (Sposito et al., 2012). This observation goes hand in hand with the hypothesized contraction of cortical representation of affected limbs, occurring after brain lesion (Liepert et al., 2000; Hallett, 2001; Flor et al., 2006; Dohle et al., 2009). We hypothesized that such a modification leads to a drift of the perceived midpoint of the limb towards the elbow in the bisection task, as if the limb was perceived shorter than it really was. Following MB training, the subjective midpoint shifts toward the hand, thus toward the objective midpoint, as if the arm was now represented longer than before the training.

This main finding suggests that even a single training session with MB can improve body representation in hemiplegic patients. This kind of short term effects are similar to those observed with other forms of embodiment, such as those experienced following a training with a tool (Sposito et al., 2012). In line with our working hypothesis the MB may have realigned the visual feedback with the patient’s intention-to-move the affected limb i.e., the will to perform a movement (Libet et al., 1983), promoting the embodiment of the mirrored healthy arm. Previous studies revealed that patients with hemiplegia show an intact intention-to-move in respect to their impaired limb (Berti et al., 2008; Fotopoulou et al., 2008; Piedimonte et al., 2016). Critically the motor command sent to the arm is not followed by any movements and the patient do not receive any visual feedback of it. Our findings are in line with the hypothesis that the MB works by inducing the realignment between the visual feedback and the patient’s intention-to-move, likely mediated by the embodiment of the reflected arm (Romano et al., 2013). This realignment may restore patients’ body representation, leading to an improvement in the detection of the arm midpoint only after MB training. Embodiment is also supported by our patients’ spontaneous subjective experience of “incorporating” the mirror-reflected arm. One of them declared “It seems that the mirror-reflected hand is the real one”, while another reported “I felt my paralyzed hand moving as the other one. That is crazy! It seemed alive”.

A second interesting result was the interaction between condition (MB/no-MB), lesion side (right/left), and chronicity (subacute/chronic). Critically chronic patients with left brain injury showed the strongest impact of MB in the body representation task, supporting the idea that these patients might be particularly suited to benefit from MB therapy. On the other hand, chronic patients with right brain injury took advantage of both motor trainings, without any specific effects of the MB.

There are several, non-mutually exclusive, explanations for these findings. First, the performance of right brain damaged patients might be influenced by Unilateral Spatial Neglect (USN). USN is the failure to report, respond, or orient to novel or meaningful personal or extrapersonal stimuli presented to the opposite side of a brain lesion. This failure cannot be attributed to either sensory or motor defects and more typically occurs after right brain injuries (Heilman et al., 1985; Vallar and Maravita, 2009). During MB training, the mirror is placed slightly off the midsagittal plane, towards the affected side, in order to make the reflection visible. So, in right brain damaged patients the reflected image may fall into the possibly neglected space. Although during the motor training attention toward the mirror was constantly suggested, this may have potentially reduced the impact of the visual feedback in patients with USN, as compared to left brain damaged patients who typically do not show neglect. We checked the presence of UNS into the medical records of our patients since the time of admission to the hospital. USN was reported in seven right brain damaged patients, equally distributed through the subacute (4) and the chronic (3) groups, suggesting that USN cannot explain, on its own, the effect that we observed.

An alternative hypothesis could be that the body schema is basically a sensory-motor representation of the body in space (Cardinali et al., 2009; de Vignemont, 2010). Its tight relation with spatial processing implicitly put it as a right hemisphere function (Devinsky, 2000), even if there is still lack of direct evidence. It is possible that left brain damaged patients have a more spared body schema, so that they are more susceptible to an experimental manipulation that involves body schema functions. Our data agree with this hypothesis, showing a selective impact of the experimental training on LBD patients, in terms of effectiveness and specificity. Indeed, a critical factor is that chronic RBD patients improved at the bisection task with both MB and no-MB training, while LBD group showed a shift towards the hand only following MB training. We can speculate about two mechanisms underlying this difference: first the LBD patients might be more sensitive to a rehabilitation technique that involves body schema functions because the critical brain side is spared by the lesion. Alternatively, the ability to code relationship between body parts, which might be lateralized in the left hemisphere (Chaminade et al., 2005; Schwoebel and Coslett, 2005), would be stimulated by the MB training, facilitating ipsilesional motor cortex excitability in LBD patients (Garry et al., 2005). Remarkably, lesion side and the chronicity of the disorder interact with each other and cannot be considered independently. Indeed, chronic patients could be affected by long lasting negative plasticity of the cortical representation of the affected arm, as well as by a more rooted learned-paralysis. Therefore, MB could show a specific effect in the LBD group, while in chronic patients we observed a generalized effect of the motor training. On the contrary, MB could enhance spontaneous recovery in subacute patients, by improving brain plasticity (Schaechter, 2004).

Future studies could better clarify the way in which different clinical features, like lesion side and the chronicity of the disorder, interact and influence MB training outcome. It is worth noting, however, that previous work has failed to correlate lesion side to the effect of MB training on motor functions (Dohle et al., 2009), so that this point needs further investigations. Moreover, we did not administer a complete neuropsychological evaluation, since we focused on motor function and body representation. So, we cannot account for any possible influences exerted by cognitive dysfunctions.

MB therapy is actually used with different clinical conditions, like phantom limb (Ramachandran and Rogers-Ramachandran, 1996), complex regional pain syndrome (McCabe et al., 2003), post-stroke motor impairments (Altschuler et al., 1999; Yavuzer et al., 2008; Dohle et al., 2009), Anton-Babinsky syndrome (Verret and Lapresle, 1978). The idea that it works by restoring the brain representation of the impaired limb is helpful to tailor more precise rehabilitation protocols on each specific patient. Following this perspective our findings have important implications about the characteristics that could influence the effectiveness of MB therapy. In clinical practice, to design customized rehabilitation plan is fundamental in order to maximize positive outcome, motivate patients and optimize resources. Moreover, a better understanding of the mechanisms underlying MB therapy could open its use in novel conditions where a body representation disorder is the core of the impairment, as recently explored for the Alien Hand Syndrome (Romano et al., 2014; Medina et al., 2015).

In conclusion, the present study suggests that MB training may positively affect body representation in patients suffering from motor impairment. Moreover, chronic patients with left brain injury seem to be more sensitive to MB training embodiment effects. Crucially, future studies using extensive MB trainings are needed in order to assess the impact of this technique on a long-lasting recovery of body representation. As well as the relation of such improvements with the recovery of motor functions. Additional studies are also required in order to evaluate different applications of this technique with other clinical conditions involving impairment of body representation, such as for the case of spinal cord injuries (Scandola et al., 2014, 2017). In addition, future investigations could better clarify the mechanisms underlying MB Therapy and the specific influence of different clinical features of the patients. It could be very interesting to correlate MB efficacy with the presence of specific cognitive and neuropsychological deficits.

## Author Contributions

All authors developed the study concept and contributed to the study design. Testing and data collection were performed by GT. DR and GT performed the data analysis and interpretation under the supervision of AM. GT drafted the manuscript, and AM and DR provided critical revisions. All authors approved the final version of the manuscript for submission.

## Conflict of Interest Statement

The authors declare that the research was conducted in the absence of any commercial or financial relationships that could be construed as a potential conflict of interest.
